# Evaluating genetic diversity differences within a likelihood framework

**DOI:** 10.1371/journal.pone.0356079

**Published:** 2026-08-14

**Authors:** David C. Nickle

**Affiliations:** 1 UW School of Global Health, Seattle, Washington, United States of America; 2 Monoceros Biosystems, San Diego, California, United States of America; Oklahoma State University, UNITED STATES OF AMERICA

## Abstract

The study of genetic diversity has a rich history, as it serves as the foundation upon which evolutionary processes act. Numerous methods have been developed to quantify genetic variation within and among populations. While some methods focus solely on allele frequencies, others assess branching patterns within phylogenetic clades without considering genetic distances. Here we introduce the **P**roportional Diversity **L**ikelihood **R**atio Statistic (PLR), a novel method that integrates genetic distances with phylogenetic tree structure to provide a comprehensive measure of genetic diversity. This method evaluates the likelihood of observed genetic data under models with constrained and unconstrained diversity, offering a robust statistical framework for testing hypotheses about genetic differentiation. The PLR approach is broadly applicable, from assessing immune repertoire diversity, such as B-cell diversity before and after vaccination, to evaluating tumor genetic diversity before and after treatment. By combining genetic distance and tree topology, PLR enables deeper insights into evolutionary dynamics and population structure. We demonstrate the use of this approach on SARS-CoV-2 sequences from two different time frames and found that diversity maybe waning. Moreover, we applied PRL to chronic lymphocytic leukemia (CLL) data, revealing that with temporally order sequences from with treatment we do not see a significant drop in diversity.

## Introduction

The concept of genetic diversity encompasses the total genetic variation within a species or a population, and understanding its distribution is crucial for evolutionary biology, conservation, and medicine. Its study has been a central focus in the field of population genetics, with significant contributions by several key figures made over the past 100 years.

One of the foundational figures in this field, Sewall Wright, made pioneering contributions through his theoretical work on population genetics. In his 1951 paper “The Genetical Structure of Populations” [1], Wright introduced the concept of the Fixation Index, or Fst, a statistical measure of population differentiation due to genetic structure. Fst has a conceptual foundation shared with ANOVA (Analysis of Variance) in that they both partition variance into components that can be attributed to different sources, i.e., populations. The Fst measure quantifies the proportion of genetic variance that can be attributed to differences among populations, relative to the total genetic variance across all populations.

Richard R. Hudson further advanced the study of genetic diversity by introducing the nearest-neighbor statistic, Snn, in 2000 [2]. Snn measures the proportion of nearest genetic neighbors in sequence space that are also nearest in geographical space. This statistic is particularly useful when genetic data consist of DNA sequences or haplotypes, and it incorporates genetic distances to assess population structure. Snn is valuable for detecting genetic differentiation, especially when allele frequencies are difficult to estimate due to high haplotype diversity or small sample sizes. By focusing on the genetic distances between sequences, Snn provides an effective way to understand the genetic relationships within and between populations, complementing traditional measures like Fst.

Key Steps in the PLR method

1. Calculate Maximum Likelihood (ML) diversity for each tree without constraints.
  a. This gives you ML estimates for the diversity of tree 1 (A) and tree 2 (B).
2. Compute the scaling factors for branch lengths.
  a. For tree 1, the scaling factor is A/ B.
  b. For tree 2, the scaling factor is B/ A.
3. Adjust branch lengths to equalized diversity.
  a. For tree 1, multiply all branch lengths by A/ B.
  b. For tree 2, multiply all branch lengths by B/ A.
4. Recalculate ML under the constraint of adjusted branch lengths.
5. This gives you new likelihoods for the constrained trees (ML_con_tree1 and ML_con_tree2).
6. Compute the likelihood ratio test statistic.
  a. The test statistic is: Λ = −2 [log(ML_con_tree1) + log(ML_con_tree2) – log(ML_tree1) – log(ML_tree2)].
7. Compare Λ to a chi-square distribution with 1 degree of freedom to determine the p-value.

Felsenstein [3] laid the groundwork for likelihood methods in phylogenetics, enabling the use of the Likelihood Ratio Test, which he applied to assess clock-like behavior in DNA sequence evolution. Goldman [4] expanded on this by using the LRT to identify the best-fitting substitution models for DNA sequence data. Building on the foundational work of Wright (Fst) and Hudson (Snn), we introduce a likelihood ratio framework that extends these concepts by incorporating both genetic distance and phylogenetic structure. We introduce a novel approach that builds on these foundational concepts. Our method utilizes the Likelihood Ratio Test (LRT) to compare genetic diversity between populations and integrates genetic distances with phylogenetic tree topology, providing a more nuanced and accurate measure of genetic diversity, with applications ranging from cancer genetics to virology. This method exemplifies the continued evolution of genetic diversity research and expands on Wright and Hudson’s work on population differentiation. Why do we need PLR? Because existing methods either ignore tree topology (π, S) or do not incorporate likelihood framework.

**Fig 1:**
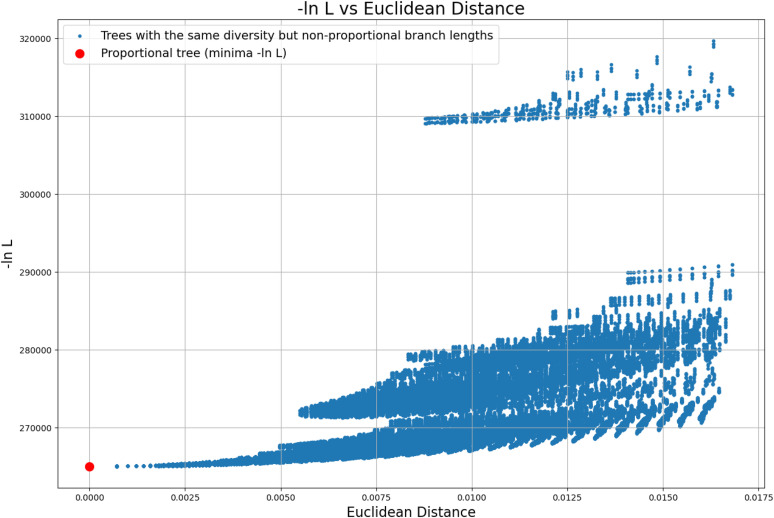
Plot of negative log-likelihood (-lnL) scores against the Euclidean distance from the proportional tree. Each blue dot represents a simulated tree, while the red dot highlights the exact proportional tree used in the simulation. The proportional tree has the smallest Euclidean distance to the original tree and achieves the maximum likelihood (lowest -lnL), demonstrating that proportional scaling produces the optimal tree for a given diversity.

The **Proportional Diversity Likelihood Ratio Statistic (PLR)** is a novel method designed to assess genetic diversity differences between populations. The PLR method begins by calculating the mean genetic diversity within each population, capturing the extent of genetic variation. This diversity measurement is then used to construct proportional trees, wherein the branch lengths are adjusted to reflect the relative differences in genetic diversity between the populations. By normalizing the diversity, these proportional trees provide a standardized framework for comparison. The core of the PLR method involves applying a Likelihood Ratio Test (LRT) to these proportional trees (PLR.R is in S1 File). The LRT evaluates whether the observed genetic diversity differences are statistically significant by comparing the likelihoods of the data under two models: one with unconstrained diversity and another with constrained average diversity (Fig 1). This integrated approach allows for a nuanced and comprehensive assessment of genetic differentiation.

### Logic for proportional tree as the ML tree

In the context of phylogenetics, diversity is typically measured as the average pairwise distance between taxa in a tree. This average distance reflects the sum of the branch lengths that connect pairs of taxa. A proportional tree is defined as a tree where the branch lengths are scaled proportionally to maintain the same relative distances between taxa. This scaling does not alter the overall topology of the tree but changes the absolute branch lengths to match a specific total diversity. The likelihood of a tree given a set of data (e.g., genetic sequences) is a measure of how well the tree explains the observed data. It is computed based on the probability of the data given the tree’s structure and branch lengths. Branch lengths in a phylogenetic tree represent evolutionary distances. They play a crucial role in determining the tree’s likelihood because they influence the probabilities of character state changes along the branches. When we scale the branch lengths proportionally to achieve a specific diversity, we are effectively preserving the relative evolutionary distances between taxa while adjusting the overall tree length. This proportional scaling maintains the integrity of the evolutionary relationships encoded in the tree. Maximum Likelihood Estimation (MLE) to find the tree (and its branch lengths) maximizes the likelihood function. In other words, it seeks the tree that best explains the observed data under the given evolutionary model. If we start with a tree that has branch lengths optimized to explain the observed data (i.e., a tree already close to the ML tree), scaling these branch lengths proportionally to match a given diversity should preserve the optimal explanation of the data. This is because the relative distances (which determine the likelihood) remain unchanged. Hence, for any given total diversity, the proportional tree is likely to remain the tree that best fits the observed data while achieving the specified diversity constraint. This makes it the ML tree for that particular diversity. Including asymmetric trees (with zero-length branches or varying branch lengths) in the analysis helps demonstrate that the proportional tree is robustly the ML tree. As you sample more trees and get closer to the proportional tree, the likelihood of these alternative trees should decrease, reinforcing the idea that the proportional tree maximizes the likelihood. Thus, the proportional tree is the most probable ML tree for any given tree and we show with simulation that the proportional tree is the ML tree constrained to a particular diversity.

## Results and discussion

For PLR to be valid the proportional tree must be the ML tree for that particular diversity. The proportional tree, by definition, adjusts the branch lengths to match a particular genetic diversity. PLR normalizes differences in diversity while preserving the overall ancestor dependent relationships depicted in the phylogenetic tree from the two populations. This normalization reflects the actual genetic distances in a way that is consistent across all populations being compared, making it an unbiased representation of the genetic diversity data at a particular level of constraint.

In the context of our Likelihood Ratio Test (LRT) approach, the maximum likelihood (ML) tree is the one that maximizes the likelihood of the observed genetic data given the model parameters. The proportional tree, by aligning branch lengths with the observed mean genetic diversity, aligns the genetic data with the most probable distribution under the assumption of uniform diversity scaling. It effectively accounts for the varying levels of diversity across sampled sequences, ensuring that the likelihood of the observed data is not disproportionately influenced by differences in diversity that are not intrinsic to the genetic structure.

To demonstrate the logic of the approach, we generated approximately 100,000 4-taxon trees, all with the same topology and total diversity but that diversity is greater than that of the true tree. These trees featured varying distributions of branch lengths constrained to the increased diversity. We tested the negative log-likelihood (−lnL) of each tree under the correct substitution model (GTR + I + G). The true tree, which represents the topology and branch length distribution used to simulate the sequence data, served as a reference for testing whether the proportional tree is consistently the maximum likelihood (ML) tree for a given diversity.

We then computed the likelihood of each tree and found the exact proportional tree to have the best likelihood. Next, we compared the likelihood scores and the Euclidean distance (a measure of the difference between the true tree and each tree with varying branch lengths). The Euclidean distance between any two trees with the same topology but different branch lengths is calculated as the straight-line distance between their corresponding vectors in this high-dimensional space. Fig 1 shows a plot of Euclidean distance against -lnL scores, clearly demonstrating that the proportional tree is the closest in terms of Euclidean distance and achieves the best -lnL score (Fig 1).

### Sequence simulation

A balanced 4-taxon tree was used to simulate sequence data under a GTR + I + G model of evolution with the following branch length pattern using SeqGen [5]:

((A:0.0125,B:0.0125):0.0125,C:0.0125,D:0.0125);

The sequence data had a total length of 150,000 bases, and the genetic diversity (π) of the tree was set to 0.05. Simulations were conducted using Seq-Gen, which generates sequences according to the specified evolutionary model and tree.

### Tree space exploration

To explore trees with the same overall diversity but varying branch lengths, we developed a custom C++ program, tree_generator (tree_generator.cpp provided in S2 File). This program generates many of the possible branch length combinations for external and internal branches, constrained by a total diversity π of 0.05. Specifically, the external branches (4 total) and internal branch (1 total) lengths were adjusted systematically in steps of 0.0005, with a strict tolerance of ±0.0005 around the target diversity. Diversity was calculated as:


$$\begin{array}{c} 𝐃𝐢𝐯𝐞𝐫𝐬𝐢𝐭𝐲\;(\pi )=[3\times 𝐒𝐮𝐦\;𝐨𝐟\;𝐄𝐱𝐭𝐞𝐫𝐧𝐚𝐥\;𝐁𝐫𝐚𝐧𝐜𝐡\;𝐋𝐞𝐧𝐠𝐭𝐡𝐬+4\times 𝐒𝐮𝐦\;𝐨𝐟\;𝐈𝐧𝐭𝐞𝐫𝐧𝐚𝐥\;𝐁𝐫𝐚𝐧𝐜𝐡\;𝐋𝐞𝐧𝐠𝐭𝐡𝐬]/4 \end{array}$$

A valid tree was one where the calculated diversity matched the target diversity within the specified tolerance. Each valid tree was written in Newick format to a file for downstream analysis.

A set of >100,000 trees was produced, all satisfying the diversity constraint.

### Likelihood estimation

The likelihood of each tree relative to the simulated sequence data was computed using PAUP* [6]. Each tree was scored under a GTR + I + G model of evolution, and the negative log-likelihood (-lnL) values were recorded.

### Euclidean distance calculation

The Euclidean distance between the starting tree (used in the Seq-Gen simulation) and each tree in the generated set was computed based on their branch lengths. For any given tree, the distance was calculated as:


Euclidean Distance=sqrt(Σ (b_i,generated−b_i,starting)^2)

where b_i represents the branch length of the i-th branch.

Python code was used for this computation, ensuring that branch lengths of both trees were aligned correctly (Euc_dist.py in S3 File).

### Data analysis and visualization

We analyzed the relationship between the Euclidean distance and the likelihood of each tree by plotting the -lnL values against the Euclidean distances. The proportional tree (where all branch lengths were scaled relative to the starting tree to match the π = 0.05 diversity) was highlighted in the plot with a red dot (Fig 1). It has the smallest Euclidean distance to the original tree and also achieves the maximum likelihood.

### PRL sensitivity and performance simulation

#### Simulation of π, s & PRL across a range of θ.

**Simulation setup:** In this simulation, we explored the relative performance of the Proportional Tree Likelihood Ratio (PRL) method alongside two traditional measures of genetic diversity, π (average pairwise distance) and S (number of segregating sites), across a range of θ values spanning from 1 × 10^-4 to 0.1. A balanced 20-taxon tree was used to maintain a consistent topology in all comparisons, and the GTR + I + G model of nucleotide substitution was employed to simulate evolutionary processes. Each simulation involved generating DNA sequences of 10,000 base pairs, with branch lengths scaled according to each θ value to vary overall genetic diversity. The resulting sequences were written to FASTA files, and π was computed for each alignment to capture average pairwise differences.

#### Analysis & null distributions.

We performed an analysis using permutation tests for both π and S, where columns of nucleotide sites were randomly shuffled between the two datasets in each comparison to generate a null distribution of observed differences where the θ would essentially be the same between the two. Significance was determined by comparing each observed difference to this null distribution. In parallel, PRL was applied by fitting an unconstrained model for each dataset, followed by evaluating the likelihood under a proportional-scaling constraint—thereby generating a likelihood ratio statistic.

#### Results and comparison of π, S, and PRL.

By comparing these three methods across multiple replicate simulations at each possible pair θ values (0.0001, 0.0002, 0.0005, 0.001, 0.002, 0.005, 0.01, 0.02, 0.05 and 0.1), we observed that PRL shows an elevated false discovery rates relative to π and S when overall diversity is extremely low (e.g., θ = 0.0001), specifically detecting differentiation when two populations share the same θ value. And it follows that PRL demonstrates superior power to detect genuine diversity differences when θ values are low but distinct between the two populations. As the ratio between θ values diverges further (e.g., < 0.1 or > 5.0), all approaches converge on detecting true differences 100% of the time. Moreover, PRL false discovery rate converges towards 0 as the length of the set of sequences gets longer. Importantly, PRL has a higher false positive rate at extremely low diversity but also higher power when diversity is truly different

## Other diversity comparing methods

### Different methods for comparing diversity between populations

There are problems with traditional methods to analyses of genetic variance. F-statistics (FST) and Nei’s Genetic Distance are a class of methods that primarily focus on genetic distance based on allele frequencies and do not consider the evolutionary relationships or tree topology. They may overlook important phylogenetic information that could influence the interpretation of genetic differentiation. AMOVA (Analysis of Molecular Variance) [7] considers hierarchical population structure, it primarily partitions genetic variance and may not fully incorporate tree topology. It provides a broad overview of genetic variance but may miss detailed evolutionary relationships. Wright’s Inbreeding Coefficients [8,9] and Diversity Indices such as Shannon’s index [10] are metrics that focus on within-population diversity or inbreeding levels without considering phylogenetic tree topology. They provide insights into genetic diversity or inbreeding but do not account for evolutionary relationships between individuals or populations. Nei and Li [11] pairwise sequence differences (π) is measures average nucleotide differences but although extremely powerful it does not take tree topology into account. It provides a measure of genetic distance but lacks context regarding how these differences are distributed across the phylogeny of ancestral dependent relationships. Similarly, the count of segregating sites (S) is a tree-independent measure that quantifies the number of sites that differ among sequences [12]. However, it operates under the infinite sites model, which assumes that every mutation occurs at a unique site and that no site is mutated more than once, a limitation that can oversimplify the complexity of real-world genetic data.

PLR Incorporating both tree topology and genetic distance, thus providing a more accurate and holistic measure of genetic differentiation between populations. By incorporating tree topology, the PLR method evaluates how genetic differences are distributed across the evolutionary tree, capturing important phylogenetic information. This allows for a better understanding of the evolutionary processes driving genetic differentiation. The PLR method avoids the pitfalls of methods that consider only one aspect (genetic distance or tree topology), providing a more robust measure that can be more sensitive to subtle differences in genetic diversity and evolutionary relationships, making it a powerful tool for detecting genetic differentiation. The PLR provides a statistical framework for hypothesis testing, allowing for rigorous evaluation of whether observed differences are statistically significant. This adds a layer of confidence to the conclusions drawn from the analysis.

The PLR method is likely a superior measure for comparing genetic diversity between populations. It provides a more comprehensive and accurate assessment of genetic differentiation by capturing both the magnitude of genetic differences and the evolutionary relationships among individuals or populations. This makes it a valuable tool for evolutionary biology, ecology, and conservation genetics.

## Examples of potential applications of PLR method in population genetics

1. A vignette: a real application to the diversity of SAR-CoV-2 (Tree and Data provided in S4 File)

To investigate the changes in SARS-CoV-2 genetic diversity over time, we applied the PLR to 2000 sequences sampled from two distinct periods: the second half of 2023 and the first half of 2024 [13] (these 2000 sequences where randomly chosen by NCBI Virus Community portal). The aim was to assess whether diversity, a proxy for effective population size, has decreased, potentially reflecting the waning of the pandemic and the impact of vaccination efforts.

Sequence data from each period were analyzed using a constrained tree model, which assumes a fixed diversity parameter, and an unconstrained model, which allows for variable diversity (Table 1).

**Table 1:** The maximum likelihood (ML) scores for each model were as follows.

Tree	Diversity (π)	Target Diversity (π)	ML Score (-lnL) uncon	ML Score (-lnL) con
2023	0.2119341	0.2045763	−106024.7	−106029.6
2024	0.1972185	0.2045763	−111327.4	−111330.7

The Likelihood Ratio Test (LRT) was performed to quantify the difference between constrained and unconstrained models, with the LRT statistic (Λ) calculated as follows:

The likelihood ratio test statistic (Λ) is calculated as follows:


$$\begin{array}{c} \Lambda =\text{2}\times [(\text{ML}\_\text{con}\_\text{2023}+\text{ML}\_\text{con}\_\text{2024})-(\text{ML}\_\text{uncon}\_\text{2023}+\text{ML}\_\text{uncon}\_\text{2024})] \end{array}$$

Substituting the values from the results:


$$\begin{array}{c} \Lambda =\text{2}\times [(-\text{111327}.\text{4}+-\text{111330}.\text{7})-(-\text{106024}.\text{7}+-\text{111327}.\text{4})] \end{array}$$

Calculate the difference:


$$\begin{array}{c} \Lambda =\text{2}\times (-\text{8}.\text{2}) \end{array}$$

$$\begin{array}{c} \Lambda =\text{16}.\text{4}\;->\text{P}-\text{value}:\;\text{5}.\text{261713e}-\text{05} \end{array}$$

This statistic was compared against a chi-square distribution with 1 degree of freedom. The resulting p-value was 5.261713e-05, indicating statistical significance at a level below 0.05. This result suggests that the constrained and unconstrained models are not equally likely, supporting the hypothesis that SARS-CoV-2 diversity has decreased between 2023 and 2024.

The observed reduction in diversity implies a potential decline in the effective population size of the virus, which is consistent with the hypothesis that widespread vaccination and other public health measures have reduced transmission rates. The decrease in diversity aligns with the notion that the pandemic is waning, as the accumulation of new mutations is less frequent relative to mutation extinction. This analysis highlights the utility of population genetic approaches, such as PLR, in monitoring the evolutionary dynamics of pathogens during and after pandemic phases.

2. A vignette: Cancer Genetics (Tree and Data provided in S4 File)

By comparing pre-treatment and post-treatment diversity in cancer cell populations one can robustly characterize a change in genetic diversity within tumors. Treatment can impose selective pressures, leading to shifts in genetic diversity. The PLR method can be used to compare genetic diversity within tumors before and after treatment and provide a more nuanced understanding of how treatment affects tumor heterogeneity.

**Fig 2:**
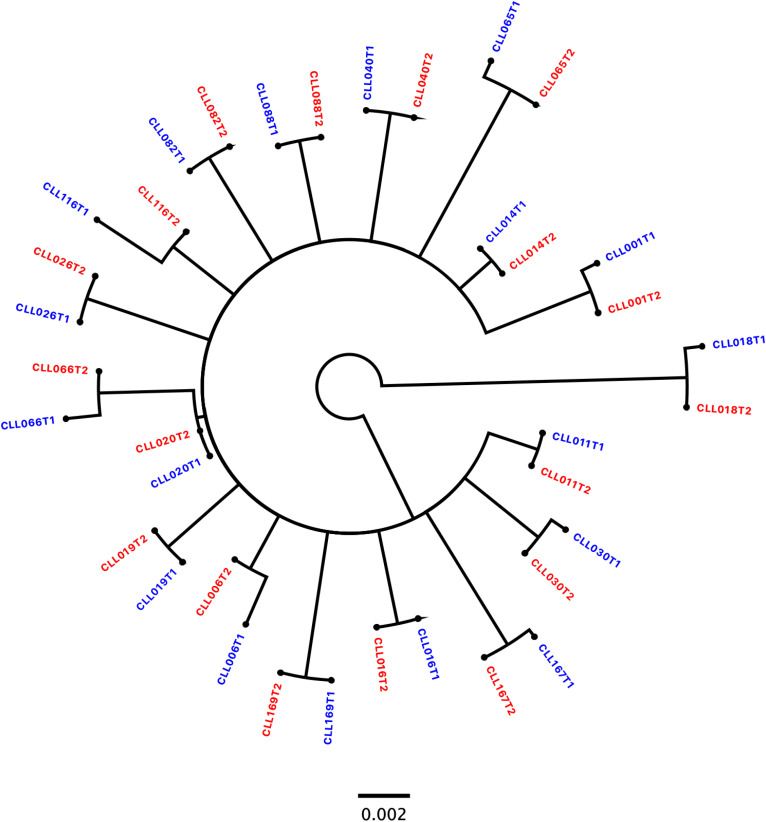
Maximum likelihood Tree estimation using Whole Exome Sequencing Data from Landau et al [14] under a GTR model of evolution using only the called segregating sites across all 18 patients with extensive sequencing. The blue labels are from Time point 1 and the red labels are from Time Point 2. Clearly the Time point 1 tips tend to reside on branches that extend further from the mid-poot root.

To that end, we obtained longitudinal mutation data from the Landau et al [14]. CLL study, parsing pre- and post-therapy samples for each patient and reconstructing variant-based sequences to perform a phylogenetic analysis. We then applied the Proportional Diversity Likelihood Ratio (PRL) method to compare genetic diversity between time points, estimating maximum-likelihood diversity values separately (unconstrained) and under a constraint of equalized branch lengths (constrained). The resulting likelihood ratio test (Λ = 1.512158) yielded a p-value of 0.2188, indicating no statistically significant difference in overall genetic diversity between the earlier and later time points. Although time point 1 had a higher raw count of mutations and thus tended to have longer branch lengths from the mid-point root (Fig 2), the PRL analysis suggests this difference is not significant in a population-genetic sense, implying that therapy did not produce a detectable reduction in clonal variation under these conditions. Of note, this interpretation is limited by bulk sequencing data: single-cell–level mutation calls would likely yield a more precise view of clonal architecture and further illuminate subtle shifts in intratumoral heterogeneity.

3. Population Genetics of Organisms

Comparing Sister Groups in the Intermountain West and the Great Plains. The Intermountain West and the Great Plains are home to sister taxa that experienced distinct historical biogeographic events during the last glaciation of North America. The Intermountain West, characterized by multiple small refuges, likely preserved greater genetic diversity in its populations. In contrast, the Great Plains, with a single large refuge, experienced reduced genetic diversity due to limited population isolation. This pattern has been observed in the *Perognathus fasciatus* species-group, where genetic diversity is influenced by these contrasting refugial histories [15].

The PLR method provides a nuanced understanding of how historical biogeographic events shape contemporary genetic diversity. Insights gained from such studies would contribute to our understanding of speciation, adaptation, and the development of effective conservation strategies.

4. Viral Genetic Diversity in HIV

Diversity Within Different Tissue Compartments in persons infected with Human Immunodeficiency Virus (HIV) can be compared using the PLR tool. HIV at time exhibits compartmentalization, where viral populations in different tissues (e.g., blood, neuronal compartments) can evolve independently. The effective population size and gene flow between compartments influence genetic diversity. The PDRLS method can compare viral genetic diversity between blood and other compartments and reveal the extent of compartmentalization and gene flow. This approach can provide insights into viral dynamics, the role of different compartments and how it relates to different disease processes.

## Conclusion

The simulation described demonstrates that the proportional tree is the maximum likelihood (ML) tree for a given diversity because proportional scaling preserves the relative evolutionary distances between taxa, which are critical for maximizing likelihood. By maintaining these relative distances while adjusting the overall tree length, the proportional tree provides the best fit to the observed data under a specified diversity constraint, emphasizing the robustness of proportional scaling for representing evolutionary relationships.

However, when simulations used relatively short sequences (e.g., ~ 1,000 base pairs), we often observed small deviations from the exact proportional tree that yielded equally high likelihoods. We attribute this to the stochastic nature of simulating limited sequence data: shorter alignments lack the statistical power to distinguish minor branch-length differences as sharply. As the simulated sequence length increases, these near-proportional alternatives lose support because the larger amount of data “smooths out” random noise, making the strictly proportional tree stand out as the single best ML solution.

Based off of 5,000 simulations over a range of diversities the PRL is more sensitive than either π or s making it more likely to detect true differences in populations when the populations have a true difference in diversity. This does come at a cost in that PRL has a higher false positive rate, meaning PRL may overestimate differences when there are none. That being said, π or s are more conservative and fail to detect true differences when the differences are real especially in the scenario when the diversities are small in both populations. Moreover, it should be noted the permutation tests for generating p values for π and s here are constrained to datasets with exactly the same number of taxa while the PRL test has no such constraint.

Our PRL analysis of longitudinal CLL samples from Landau et al. found no statistically significant difference in overall genetic diversity between pre- and post-therapy time points (p = 0.2188), despite a higher raw mutation count at the earlier time point. This outcome suggests that the treatment studied did not visibly reduce clonal variation under the conditions examined. However, PRL—like any statistical test—does not always reject the null hypothesis as suggested by the false discovery rate, so, a negative result may stem from limited power or subtle changes masked by bulk sequencing. Single-cell data, by contrast, would likely provide a more nuanced view of clonal shifts and intratumoral heterogeneity.

When applying the PRL method to SARS-CoV-2 sequences, we observed a statistically significant decrease in diversity between the second half of 2023 and the first half of 2024. The likelihood ratio test (LRT) supports this finding, with a p-value of 0.00715, suggesting that the constrained and unconstrained models are not equally likely. This decrease in diversity may indicate a reduction in the effective population size of the virus, potentially due to the combined effects of vaccination and other public health measures. Understanding this trend has significant implications for monitoring the pandemic’s trajectory and assessing the long-term impact of interventions on viral evolution and transmission. These results, however, are dependent on the vagaries of NCBI Virus portal delivering a random set of sequences that are representative of the actual pandemic.

## Supporting information

S1 File PLR.R Is the code to computer PRL with Two Trees and Two DNA sequences datasets. (ZIP)

S2 File tree_generator.cpp Is the code to generate 4 taxon trees under a given diversity. (ZIP)

S3 File Euc_dist.py Is the code to compute euclidian distance between trees. (ZIP)

S4 File Sequences and Trees used in the two vignettes. (ZIP)

S5 File Fig S power plots. (PDF)

S6 File Power comparison PLR Snn Fst. (R)
