# Supplementary material for: Evaluating genetic diversity differences within a likelihood framework
Source: PLoS One. 2026 Aug 14;21(8):e0356079. doi: 10.1371/journal.pone.0356079 (PMC13475874; doi:10.1371/journal.pone.0356079)
Supplement: Supplementary file 1 [file pone.0356079.s005.pdf]

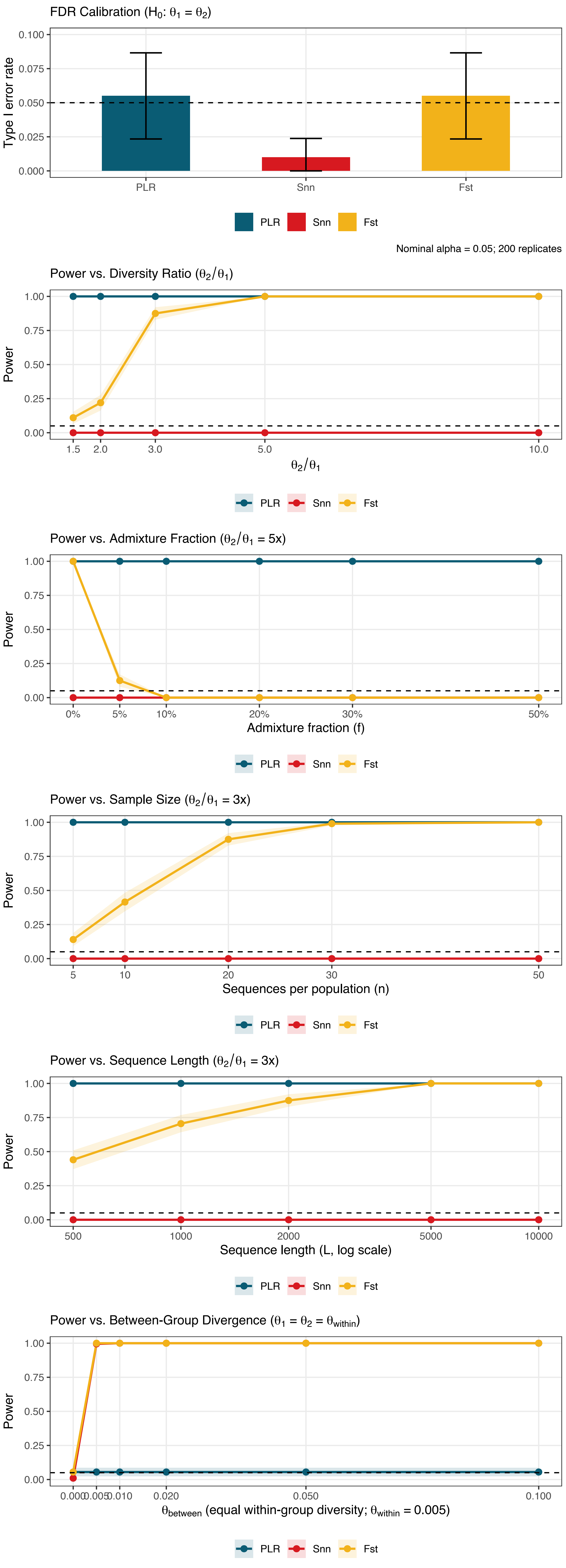

FDR Calibration (H_0: $\theta_1 = \theta_2$)
Type I error rate
0.100
0.075
0.050
0.025
0.000
PLR
Snn
Fst
PLR
Snn
Fst
Nominal alpha = 0.05; 200 replicates
Power vs. Diversity Ratio (θ_2/θ_1)
Power
1.00
0.75
0.50
0.25
0.00
1.5
2.0
3.0
5.0
10.0
θ_2/θ_1
PLR
Snn
Fst
Power vs. Admixture Fraction (θ_2/θ_1 = 5x)
Power
1.00
0.75
0.50
0.25
0.00
0%
5%
10%
20%
30%
50%
Admixture fraction (f)
PLR
Snn
Fst
Power vs. Sample Size (θ_2/θ_1 = 3x)
Power
1.00
0.75
0.50
0.25
0.00
5
10
20
30
50
Sequences per population (n)
PLR
Snn
Fst
Power vs. Sequence Length (θ_2/θ_1 = 3x)
Power
1.00
0.75
0.50
0.25
0.00
500
1000
2000
5000
10000
Sequence length (L, log scale)
PLR
Snn
Fst
Power vs. Between-Group Divergence ($\theta_1 = \theta_2 = \theta_{within}$)
Power
1.00
0.75
0.50
0.25
0.00
0.000
0.005
0.010
0.020
0.050
0.100
$\theta_{between}$ (equal within-group diversity; θ_{within} = 0.005)
PLR
Snn
Fst
