# Supplementary material for: Evaluating genetic diversity differences within a likelihood framework
Source: PLoS One. 2026 Aug 14;21(8):e0356079. doi: 10.1371/journal.pone.0356079 (PMC13475874; doi:10.1371/journal.pone.0356079)
Supplement: Supplementary file 2 [file pone.0356079.s008.docx]

March 18, 2026

Editorial Office, PLOS ONE

Re: Manuscript PONE-D-25-04146 — *Evaluating genetic diversity differences within a likelihood framework*

Revision submission

Dear Dr. Stephens and editorial team,

We thank the Academic Editor and both reviewers for the detailed evaluation of our manuscript. The comments have substantially strengthened the work. We address each point below with specific reference to the changes made in the revised manuscript. All modifications are indicated by tracked changes in the accompanying “Revised Manuscript with Track Changes” file.

The two most substantial revisions are: (1) the addition of a six-panel power comparison of PLR against Hudson’s S_nn_ and the Hudson–Slatkin–Maddison F_ST_ estimator, with a new supplementary figure (Figure S_power) and complete simulation code (Supporting Information S5); and (2) a thorough reframing of PLR’s inferential scope relative to phylogenetic diversity metrics including Faith’s PD. Additional revisions address hypothesis statement clarity, a concrete motivating scenario, mathematical justification of the proportional-tree assumption, and replacement of subjective comparative language with quantitative claims from the simulations.

**Response to the Academic Editor**

The editor requested that we clarify how PLR relates to Faith’s phylogenetic diversity (PD) and related community-level metrics, citing Faith (1992) and Fountain-Jones et al. (2022) as touchstones. We have added a dedicated paragraph to the Results and Discussion that explicitly distinguishes the two frameworks. Faith’s PD is an absolute measure of the total branch length spanning a set of taxa, widely applied in community ecology and conservation biology to quantify biodiversity across species. PLR is a statistical test for whether two samples drawn from the same or related species differ in within-population genetic diversity (θ₁ vs. θ₂)—a fundamentally different inferential question at the intraspecific rather than interspecific scale. Fountain-Jones et al. (2022) is cited to illustrate how tree-based approaches have been applied to intraspecific viral diversity dynamics, contextualizing PLR within that line of inquiry. Both references have been added to the reference list (refs. 8 and 9).

We agree with the editor’s assessment that the simulation work is a strength. We have retained the core proportional-tree validation in the main text and placed the extended power comparison in supplementary material with a summary subsection (“Power comparison: PLR vs. S_nn_ vs. F_ST_”) in the main text.

**Response to Reviewer #1**

We thank Reviewer #1 for a thorough and technically precise critique. We address each substantive point below.

**Comment 1.1 — Null and alternative hypotheses; definition of constrained and unconstrained models.**

*The reviewer requested a clear statement of the null and alternative hypotheses and unambiguous definitions of the terms “constrained” and “unconstrained” throughout the manuscript.*

**Response:** The Introduction now contains an explicit paragraph stating the formal null hypothesis H₀: θ₁ = θ₂ (the two populations share identical mean genetic diversity) against H₁: θ₁ ≠ θ₂. The paragraph defines “unconstrained model” as the model in which each population’s phylogenetic tree retains its own maximum-likelihood branch lengths, yielding independent ML scores, and “constrained model” as the model in which both trees are rescaled to a common target diversity (the arithmetic mean of the two observed diversities) via proportional branch-length adjustment. This is the ML tree subject to the constraint θ₁ = θ₂. A large likelihood ratio statistic Λ indicates that the scaling constraint substantially reduces fit, supporting rejection of H₀.

**Comment 1.2 — Motivating scenario.**

*The reviewer suggested grounding the method in a concrete biological scenario to aid reader orientation.*

**Response:** The Introduction now includes a motivating scenario: two samples collected from geographically separated populations—one from a small refugial habitat with limited effective population size, one from an open habitat with large effective population size. The scenario explains why a researcher would want to test whether the two populations differ in genetic diversity (a proxy for N_e_ and evolutionary history), and why PLR offers additional discriminatory power over π and S when tree shapes differ even at similar scalar diversity levels.

**Comment 1.3 — Mathematical justification of the proportional-tree assumption.**

*The reviewer noted that simulation alone cannot prove the proportional tree is the ML tree for a given diversity, and suggested adding justification from the mathematics of the models themselves.*

**Response:** The “Logic for Proportional Tree as the ML Tree” section now contains an analytical justification alongside the simulation. Under the GTR family, the likelihood depends on branch lengths solely through expected substitution counts per site. Let T* denote the ML tree with log-likelihood ℓ(T*). Any reparameterization cT* (all branches multiplied by c > 0) preserves substitution-rate ratios, so the likelihood surface in the direction of uniform scaling is smooth and unimodal: no reallocation of branch length improves likelihood above cT*. Consequently, for any prescribed diversity π’, the proportional rescaling of T* is the constrained ML tree. The numerical experiments across >100,000 four-taxon configurations corroborate this analytically motivated result.

**Comment 1.4 — Power comparison against Snn and FST.**

*The reviewer requested a quantitative comparison of PLR’s statistical power against established population-differentiation statistics, with tabular or graphical results and clear simulation parameters, rather than subjective assertions about PLR’s superiority.*

**Response:** We have added the subsection “Power comparison: PLR vs. S_nn_ vs. F_ST_” and Supplementary Figure S_power (six panels). Simulations used n = 20 sequences per population and L = 2,000 bp under a Jukes–Cantor model unless otherwise varied; all power estimates are based on 200 replicates. Significance for S_nn_ and F_ST_ was assessed by 200-replicate permutation tests; PLR significance by the χ²(1) asymptotic approximation.

The six panels address: (1) false positive rate calibration under the null (θ₁ = θ₂); (2) power as a function of diversity ratio θ₂/θ₁ from 1.5× to 10×; (3) power as a function of admixture fraction at a fixed 5× diversity ratio; (4) power as a function of sample size n = 5–50; (5) power as a function of sequence length L = 500–10,000 bp; and (6) power when populations are differentiated in allele-frequency composition but share equal within-group diversity.

Key results: all three tests are well-calibrated at α = 0.05 under the null (panel 1). When within-population diversities differ, PLR substantially outperforms FST across diversity ratios of 1.5× to 10× (panel 2); notably, Snn has essentially no power in this scenario because it tests nearest-neighbor clustering rather than diversity magnitude—a result that underscores the complementarity of the three statistics. PLR maintains high power even under moderate admixture between populations (panel 3), and reaches 80% power at smaller sample sizes and shorter sequence lengths than FST (panels 4 and 5). Conversely, when populations are differentiated in allele-frequency composition but share equal within-group diversity, Snn and FST gain power as expected while PLR remains at the false positive rate (panel 6). This complementarity is discussed explicitly in the revised text.

We have replaced all subjective comparative language (“more nuanced and accurate,” “likely a superior measure”) with quantitative claims grounded in these simulation results. The revised text now states that PLR achieves greater power than S_nn_ or F_ST_ specifically when the inferential target is a difference in within-population diversity (θ₁ ≠ θ₂), while S_nn_ and F_ST_ are the appropriate tools when the question concerns allele-frequency differentiation at equal diversity. PLR is framed throughout as a complement to, not a replacement for, existing statistics.

**Comment 1.5 — Simulation parameters for differentiated populations.**

*The reviewer asked for clarity on what “differentiated populations” means in the sensitivity simulation context.*

**Response:** The power comparison subsection now specifies that “differentiated populations” in the simulation context means two independent samples generated from a shared ancestral sequence under a Jukes–Cantor model with distinct θ values (θ₁ ≠ θ₂), with no gene flow between them. The six-panel figure caption and the methods text both specify default parameters (n = 20, L = 2,000 bp, θ₁ = 0.005) and the dimension varied in each panel. Complete simulation code is provided in Supporting Information S5.

**Response to Reviewer #2**

We thank Reviewer #2 for the positive assessment and for the specific recommendations.

**Comment 2.1 — Sentence revision: “sources, i.e., populations.”**

*The reviewer requested the sentence be rewritten to read “sources such as populations” rather than “sources, i.e., populations.”*

**Response:** Corrected. The sentence in the F_ST_ paragraph of the Introduction now reads: “F_ST_ has a conceptual foundation shared with ANOVA in that they both partition variance into components attributed to different sources such as populations.”

**Comment 2.2 — Relationship to Faith’s PD and inferential scope.**

*The reviewer raised concerns about adequately distinguishing PLR from Faith’s phylogenetic diversity and clarifying that PLR is a test for equal within-population diversity, not a general population-differentiation test.*

**Response:** A paragraph has been added to the Results and Discussion that explicitly distinguishes PLR from Faith’s PD (Faith 1992). Faith’s PD quantifies the total branch length of a phylogeny spanning a set of taxa—an absolute measure of evolutionary breadth applied in community ecology and conservation biology across species. PLR is a statistical test for whether two samples from the same or related species differ in within-population genetic diversity (θ₁ = θ₂ vs. θ₁ ≠ θ₂)—a fundamentally different inferential question. Fountain-Jones et al. (2022) is cited to contextualize tree-based approaches to intraspecific diversity dynamics, within which PLR provides a formal likelihood-based test rather than a descriptive index.

The abstract, Introduction, and Discussion have been revised throughout to make explicit that PLR’s null hypothesis is equal within-population diversity, distinct from the null of no allele-frequency differentiation tested by F_ST_ and S_nn_. Panel 6 of the power comparison provides empirical support: PLR has no power to detect allele-frequency differentiation when diversity levels are equal, confirming that it is not a drop-in replacement for existing population-differentiation tests.

**Journal Requirement Checklist**

1. Style requirements: The revised manuscript has been checked against PLOS ONE style templates.

2. ORCID iD: The corresponding author’s ORCID (0000-0002-8672-6261) is validated in Editorial Manager.

3. Code sharing: All author-generated code underpinning the findings is provided in Supporting Information S1 (PLR.R), S2 (tree_generator.cpp), S3 (Euc_dist.py), and S5 (power_comparison_PLR_Snn_Fst.R), without restriction.

4. Funding: Funding text has been removed from the manuscript body. Funding information appears only in the Funding Statement section of the submission form.

5. Recommended citations: Faith (1992) and Fountain-Jones et al. (2022) have been reviewed, found directly relevant, and incorporated into the manuscript with substantive discussion (refs. 8 and 9).

We hope the revised manuscript is now suitable for publication. We are happy to provide additional clarification if needed.

Sincerely,

David C. Nickle

dnickle@uw.edu
