# Supplementary material for: Evaluating genetic diversity differences within a likelihood framework
Source: PLoS One. 2026 Aug 14;21(8):e0356079. doi: 10.1371/journal.pone.0356079 (PMC13475874; doi:10.1371/journal.pone.0356079)
Supplement: Peer review history

# Peer review: Decision Letter 0

**DOI:** 10.1371/journal.pone.0356079.r001

**Authors:** Patrick Stephens

8 Sep 2025

Dear Dr. Nickle,

Thank you for submitting your manuscript to PLOS ONE. After careful consideration, we feel that it has merit but does not fully meet PLOS ONE’s publication criteria as it currently stands. Therefore, we invite you to submit a revised version of the manuscript that addresses the points raised during the review process.

Please submit your revised manuscript by Oct 23 2025 11:59PM. If you will need more time than this to complete your revisions, please reply to this message or contact the journal office at plosone@plos.org

- A rebuttal letter that responds to each point raised by the academic editor and reviewer(s). You should upload this letter as a separate file labeled 'Response to Reviewers'.
- A marked-up copy of your manuscript that highlights changes made to the original version. You should upload this as a separate file labeled 'Revised Manuscript with Track Changes'.
- An unmarked version of your revised paper without tracked changes. You should upload this as a separate file labeled 'Manuscript'.

If you would like to make changes to your financial disclosure, please include your updated statement in your cover letter. Guidelines for resubmitting your figure files are available below the reviewer comments at the end of this letter.

If applicable, we recommend that you deposit your laboratory protocols in protocols.io to enhance the reproducibility of your results. Protocols.io assigns your protocol its own identifier (DOI) so that it can be cited independently in the future. For instructions see: https://journals.plos.org/plosone/s/submission-guidelines#loc-laboratory-protocols. Additionally, PLOS ONE offers an option for publishing peer-reviewed Lab Protocol articles, which describe protocols hosted on protocols.io. Read more information on sharing protocols at https://plos.org/protocols?utm_medium=editorial-email&utm_source=authorletters&utm_campaign=protocols.

We look forward to receiving your revised manuscript.

Kind regards,

Patrick R Stephens, Ph.D.

Academic Editor

PLOS ONE

Cited in my comments:

*Biological conservation* , *61* (1), 1-10.

Fountain-Jones, N. M., Kraberger, S., Gagne, R. B., Gilbertson, M. L., Trumbo, D. R., Charleston, M., ... & Craft, M. E. (2022). Hunting alters viral transmission and evolution in a large carnivore. *Nature ecology & evolution* , *6*

Journal Requirements:

When submitting your revision, we need you to address these additional requirements.

1.Please ensure that your manuscript meets PLOS ONE's style requirements, including those for file naming. The PLOS ONE style templates can be found at

https://journals.plos.org/plosone/s/file?id=wjVg/PLOSOne_formatting_sample_main_body.pdf and

https://journals.plos.org/plosone/s/file?id=ba62/PLOSOne_formatting_sample_title_authors_affiliations.pdf

2. PLOS requires an ORCID iD for the corresponding author in Editorial Manager on papers submitted after December 6th, 2016. Please ensure that you have an ORCID iD and that it is validated in Editorial Manager. To do this, go to ‘Update my Information’ (in the upper left-hand corner of the main menu), and click on the Fetch/Validate link next to the ORCID field. This will take you to the ORCID site and allow you to create a new iD or authenticate a pre-existing iD in Editorial Manager.

3. Please note that PLOS ONE has specific guidelines on code sharing for submissions in which author-generated code underpins the findings in the manuscript. In these cases, we expect all author-generated code to be made available without restrictions upon publication of the work. Please review our guidelines at https://journals.plos.org/plosone/s/materials-and-software-sharing#loc-sharing-code and ensure that your code is shared in a way that follows best practice and facilitates reproducibility and reuse.

4. Please note that funding information should not appear in any section or other areas of your manuscript. We will only publish funding information present in the Funding Statement section of the online submission form. Please remove any funding-related text from the manuscript.

5. If the reviewer comments include a recommendation to cite specific previously published works, please review and evaluate these publications to determine whether they are relevant and should be cited. There is no requirement to cite these works unless the editor has indicated otherwise.

Additional Editor Comments:

First off, please accept my apologies on behalf of the journal for how long this process took.  As you are aware, I was only brought in as a handling editor after someone else had been overseeing this article for months.  However, even once I took over, it took much longer than I would have liked to secure these reviews and I thank you for your patience.

We have somewhat of a split decision.  However, I find that I agree with most of the feedback of reviewer two, who is eminently qualified to critique this work.  In addition, there are a host of diversity metrics that incorporate phylogeny, such as Faith's phylogenetic diversity (Faith 1992).  These metrics have only relatively recently begun to be applied in the field of viral phylodynamics (e.g., Fountain-Jones 2022), so to me a hybrid approach that combines aspects of both Faith's PD and within-species genetic diversity indices is fairly exciting (full citations below).  In addition to the suggestions of reviewer two, clarifying the relationship of your new metric to those much older community level metrics might also be useful.

I find the simulation work to be a strength of the study, so I leave it entirely to your discretion whether to keep it in the main text or relegate it to the supplement.  Regardless, I completely agree with reviewer two that the motivation for this new measure and its characteristics compared to existing measures need to be made clearer for readers.

[Note: HTML markup is below. Please do not edit.]

Reviewer's Responses to Questions

**Comments to the Author**

1. Is the manuscript technically sound, and do the data support the conclusions?

Reviewer #1: Partly

Reviewer #2: Yes

**********

2. Has the statistical analysis been performed appropriately and rigorously?

Reviewer #1: No

Reviewer #2: Yes

**********

3. Have the authors made all data underlying the findings in their manuscript fully available?

The PLOS Data policy

Reviewer #1: Yes

Reviewer #2: Yes

**********

4. Is the manuscript presented in an intelligible fashion and written in standard English?

Reviewer #1: Yes

Reviewer #2: Yes

**********

Reviewer #1: The author proposes a potentially novel statistical method for comparing the observed nucleotide diversities in a given genomic region between two nominal populations of individuals. The intent of the method is presumably to include estimated phylogenies of the two populations in the comparison, which is hypothesized to increase the power to detect diversity differences over other statistics (such as pi and S) which essentially average with equal weight over all possible phylogenies.

I confess to having had a difficult time following the motivation and description of the method. I would like to see a very clear statement of the null and alternative hypotheses that are being compared. The terms “constrained” and “unconstrained” need more clear definition in the context of the test. It isn’t always clear to me that the terms “dataset”, “sample”, and “population” mean the same thing everywhere they are mentioned. A simple scenario that could motivate the whole effort might be the following: “Consider two sets of individuals, one set sampled from one geographic location, the other from another location. Can we infer from differences in genetic diversity that the samples represent independently evolving, genetically isolated populations?” and referring back to this scenario in describing the method.

The intuition is sound that the shape of the phylogeny can distort the distributions of population genetic statistics in a detectable way. For example, Tajima’s (1992[ ?]) D examines the difference pi - S, which has zero as the expected value under the neutral null hypothesis, but skews negative if internal branches are short compared to leaf branches (e.g., under a recent selective sweep).

There is much effort expended convincing the reader that, if a ML phylogeny, call it T*, is inferred based on a given set of sequences, then a phylogeny “cT*”, obtained by multiplying all branch lengths in T* by some constant c, maximizes the likelihood given the sequences over all other phylogenies with the same total scaled branch length. I believe this conjecture is reasonable, but a single simulation can’t prove it. I would mention the results of the simulation in the paper, but relegate the details to supplementary material. A better approach would be to add a justification (not necessarily a proof) from the mathematics of the models themselves.

While the author suggests that the method provides “a more nuanced and accurate measure of genetic diversity” and similar language, this sort of description is subjective and not quantitative. The proposed method is still a univariate statistic that subsumes its inputs under a null model of evolution, like the other statistics mentioned. And, it may “work” regardless of how accurate the “proportional tree assumption” above is. Therefore, a more compelling analysis to me would compare the performances of the proposed statistic, Snn, and Fst to detect population differentiation between simulated samples from two populations over varying degrees of admixture, for example. If the new statistic has more power than the standard statistics to detect population differentiation - for a given level of diversity (mutation rate), sample size, region length, gene flow, or other variables- then this would be more compelling reason to use the statistic than just asserting that it explicitly incorporates phylogeny. Basically, I would like to see an expanded version of the section “PRL Sensitivity and Performance Simulation”, with tables of results comparing the proposed statistic with at least one of the earlier population differentiation statistics, with more detail about the simulated “differentiated populations” mentioned in that section. (I assume these are two samples generated independently (i.e., two completely isolated populations), but this isn’t totally clear in the text.)

To summarize, the proposed statistic is interesting, and a revision should concentrate on demonstrating whether it is quantitatively better than existing statistics, rather than overthinking a reasonable assumption but assuming that the reader will buy into a subjective argument about trees.

Reviewer #2: The article “Evaluating genetic diversity differences within a likelihood framework” is well written and provides an overview that led to the development of the Proportional Diversity Likelihood Ratio Statistic (PLRs).

The author should rewrite this sentence in the introduction section to read as follows;

….sources, i.e., populations…Rewrite to… sources such as populations.

**********

what does this mean?). If published, this will include your full peer review and any attached files.

If you choose “no”, your identity will remain anonymous but your review may still be made public.

**Do you want your identity to be public for this peer review?**  For information about this choice, including consent withdrawal, please see our Privacy Policy

Reviewer #1: No

Reviewer #2: No

**********

[NOTE: If reviewer comments were submitted as an attachment file, they will be attached to this email and accessible via the submission site. Please log into your account, locate the manuscript record, and check for the action link "View Attachments". If this link does not appear, there are no attachment files.]

While revising your submission, please upload your figure files to the Preflight Analysis and Conversion Engine (PACE) digital diagnostic tool, https://pacev2.apexcovantage.com/. PACE helps ensure that figures meet PLOS requirements. To use PACE, you must first register as a user. Registration is free. Then, login and navigate to the UPLOAD tab, where you will find detailed instructions on how to use the tool. If you encounter any issues or have any questions when using PACE, please email PLOS at figures@plos.org. Please note that Supporting Information files do not need this step.

# Author comment: Author response to Decision Letter 1

**DOI:** 10.1371/journal.pone.0356079.r002

8 Jun 2026

March 18, 2026

Editorial Office, PLOS ONE

Re: Manuscript PONE-D-25-04146 — Evaluating genetic diversity differences within a likelihood framework

Revision submission

Dear Dr. Stephens and editorial team,

We thank the Academic Editor and both reviewers for the detailed evaluation of our manuscript. The comments have substantially strengthened the work. We address each point below with specific reference to the changes made in the revised manuscript. All modifications are indicated by tracked changes in the accompanying “Revised Manuscript with Track Changes” file.

The two most substantial revisions are: (1) the addition of a six-panel power comparison of PLR against Hudson’s Snn and the Hudson–Slatkin–Maddison FST estimator, with a new supplementary figure (Figure S_power) and complete simulation code (Supporting Information S5); and (2) a thorough reframing of PLR’s inferential scope relative to phylogenetic diversity metrics including Faith’s PD. Additional revisions address hypothesis statement clarity, a concrete motivating scenario, mathematical justification of the proportional-tree assumption, and replacement of subjective comparative language with quantitative claims from the simulations.

Response to the Academic Editor

The editor requested that we clarify how PLR relates to Faith’s phylogenetic diversity (PD) and related community-level metrics, citing Faith (1992) and Fountain-Jones et al. (2022) as touchstones. We have added a dedicated paragraph to the Results and Discussion that explicitly distinguishes the two frameworks. Faith’s PD is an absolute measure of the total branch length spanning a set of taxa, widely applied in community ecology and conservation biology to quantify biodiversity across species. PLR is a statistical test for whether two samples drawn from the same or related species differ in within-population genetic diversity (θ₁ vs. θ₂)—a fundamentally different inferential question at the intraspecific rather than interspecific scale. Fountain-Jones et al. (2022) is cited to illustrate how tree-based approaches have been applied to intraspecific viral diversity dynamics, contextualizing PLR within that line of inquiry. Both references have been added to the reference list (refs. 8 and 9).

We agree with the editor’s assessment that the simulation work is a strength. We have retained the core proportional-tree validation in the main text and placed the extended power comparison in supplementary material with a summary subsection (“Power comparison: PLR vs. Snn vs. FST”) in the main text.

Response to Reviewer #1

We thank Reviewer #1 for a thorough and technically precise critique. We address each substantive point below.

Comment 1.1 — Null and alternative hypotheses; definition of constrained and unconstrained models.

The reviewer requested a clear statement of the null and alternative hypotheses and unambiguous definitions of the terms “constrained” and “unconstrained” throughout the manuscript.

Response: The Introduction now contains an explicit paragraph stating the formal null hypothesis H₀: θ₁ = θ₂ (the two populations share identical mean genetic diversity) against H₁: θ₁ ≠ θ₂. The paragraph defines “unconstrained model” as the model in which each population’s phylogenetic tree retains its own maximum-likelihood branch lengths, yielding independent ML scores, and “constrained model” as the model in which both trees are rescaled to a common target diversity (the arithmetic mean of the two observed diversities) via proportional branch-length adjustment. This is the ML tree subject to the constraint θ₁ = θ₂. A large likelihood ratio statistic Λ indicates that the scaling constraint substantially reduces fit, supporting rejection of H₀.

Comment 1.2 — Motivating scenario.

The reviewer suggested grounding the method in a concrete biological scenario to aid reader orientation.

Response: The Introduction now includes a motivating scenario: two samples collected from geographically separated populations—one from a small refugial habitat with limited effective population size, one from an open habitat with large effective population size. The scenario explains why a researcher would want to test whether the two populations differ in genetic diversity (a proxy for Ne and evolutionary history), and why PLR offers additional discriminatory power over π and S when tree shapes differ even at similar scalar diversity levels.

Comment 1.3 — Mathematical justification of the proportional-tree assumption.

The reviewer noted that simulation alone cannot prove the proportional tree is the ML tree for a given diversity, and suggested adding justification from the mathematics of the models themselves.

Response: The “Logic for Proportional Tree as the ML Tree” section now contains an analytical justification alongside the simulation. Under the GTR family, the likelihood depends on branch lengths solely through expected substitution counts per site. Let T* denote the ML tree with log-likelihood ℓ(T*). Any reparameterization cT* (all branches multiplied by c > 0) preserves substitution-rate ratios, so the likelihood surface in the direction of uniform scaling is smooth and unimodal: no reallocation of branch length improves likelihood above cT*. Consequently, for any prescribed diversity π’, the proportional rescaling of T* is the constrained ML tree. The numerical experiments across >100,000 four-taxon configurations corroborate this analytically motivated result.

Comment 1.4 — Power comparison against Snn and FST.

The reviewer requested a quantitative comparison of PLR’s statistical power against established population-differentiation statistics, with tabular or graphical results and clear simulation parameters, rather than subjective assertions about PLR’s superiority.

Response: We have added the subsection “Power comparison: PLR vs. Snn vs. FST” and Supplementary Figure S_power (six panels). Simulations used n = 20 sequences per population and L = 2,000 bp under a Jukes–Cantor model unless otherwise varied; all power estimates are based on 200 replicates. Significance for Snn and FST was assessed by 200-replicate permutation tests; PLR significance by the χ²(1) asymptotic approximation.

The six panels address: (1) false positive rate calibration under the null (θ₁ = θ₂); (2) power as a function of diversity ratio θ₂/θ₁ from 1.5× to 10×; (3) power as a function of admixture fraction at a fixed 5× diversity ratio; (4) power as a function of sample size n = 5–50; (5) power as a function of sequence length L = 500–10,000 bp; and (6) power when populations are differentiated in allele-frequency composition but share equal within-group diversity.

Key results: all three tests are well-calibrated at α = 0.05 under the null (panel 1). When within-population diversities differ, PLR substantially outperforms FST across diversity ratios of 1.5× to 10× (panel 2); notably, Snn has essentially no power in this scenario because it tests nearest-neighbor clustering rather than diversity magnitude—a result that underscores the complementarity of the three statistics. PLR maintains high power even under moderate admixture between populations (panel 3), and reaches 80% power at smaller sample sizes and shorter sequence lengths than FST (panels 4 and 5). Conversely, when populations are differentiated in allele-frequency composition but share equal within-group diversity, Snn and FST gain power as expected while PLR remains at the false positive rate (panel 6). This complementarity is discussed explicitly in the revised text.

We have replaced all subjective comparative language (“more nuanced and accurate,” “likely a superior measure”) with quantitative claims grounded in these simulation results. The revised text now states that PLR achieves greater power than Snn or FST specifically when the inferential target is a difference in within-population diversity (θ₁ ≠ θ₂), while Snn and FST are the appropriate tools when the question concerns allele-frequency differentiation at equal diversity. PLR is framed throughout as a complement to, not a replacement for, existing statistics.

Comment 1.5 — Simulation parameters for differentiated populations.

The reviewer asked for clarity on what “differentiated populations” means in the sensitivity simulation context.

Response: The power comparison subsection now specifies that “differentiated populations” in the simulation context means two independent samples generated from a shared ancestral sequence under a Jukes–Cantor model with distinct θ values (θ₁ ≠ θ₂), with no gene flow between them. The six-panel figure caption and the methods text both specify default parameters (n = 20, L = 2,000 bp, θ₁ = 0.005) and the dimension varied in each panel. Complete simulation code is provided in Supporting Information S5.

Response to Reviewer #2

We thank Reviewer #2 for the positive assessment and for the specific recommendations.

Comment 2.1 — Sentence revision: “sources, i.e., populations.”

The reviewer requested the sentence be rewritten to read “sources such as populations” rather than “sources, i.e., populations.”

Response: Corrected. The sentence in the FST paragraph of the Introduction now reads: “FST has a conceptual foundation shared with ANOVA in that they both partition variance into components attributed to different sources such as populations.”

Comment 2.2 — Relationship to Faith’s PD and inferential scope.

The reviewer raised concerns about adequately distinguishing PLR from Faith’s phylogenetic diversity and clarifying that PLR is a test for equal within-population diversity, not a general population-differentiation test.

Response: A paragraph has been added to the Results and Discussion that explicitly distinguishes PLR from Faith’s PD (Faith 1992). Faith’s PD quantifies the total branch length of a phylogeny spanning a set of taxa—an absolute measure of evolutionary breadth applied in community ecology and conservation biology across species. PLR is a statistical test for whether two samples from the same or related species differ in within-population genetic diversity (θ₁ = θ₂ vs. θ₁ ≠ θ₂)—a fundamentally different inferential question. Fountain-Jones et al. (2022) is cited to contextualize tree-based approaches to intraspecific diversity dynamics, within which PLR provides a formal likelihood-based test rather than a descriptive index.

The abstract, Introduction, and Discussion have been revised throughout to make explicit that PLR’s null hypothesis is equal within-population diversity, distinct from the null of no allele-frequency differentiation tested by FST and Snn. Panel 6 of the power comparison provides empirical support: PLR has no power to detect allele-frequency differentiation when diversity levels are equal, confirming that it is not a drop-in replacement for existing population-differentiation tests.

Journal Requirement Checklist

1. Style requirements: The revised manuscript has been checked against PLOS ONE style templates.

2. ORCID iD: The corresponding author’s ORCID (0000-0002-8672-6261) is validated in Editorial Manager.

3. Code sharing: All author-generated code underpinning the findings is provided in Supporting Information S1 (PLR.R), S2 (tree_generator.cpp), S3 (Euc_dist.py), and S5 (power_comparison_PLR_Snn_Fst.R), without restriction.

4. Funding: Funding text has been removed from the manuscript body. Funding information appears only in the Funding Statement section of the submission form.

5. Recommended citations: Faith (1992) and Fountain-Jones et al. (2022) have been reviewed, found directly relevant, and incorporated into the manuscript with substantive discussion (refs. 8 and 9).

Attachment Submitted filename: Reply_to_Reviewers_PONE-D-25-04146_v3.docx

# Peer review: Decision Letter 1

**DOI:** 10.1371/journal.pone.0356079.r003

**Authors:** Patrick Stephens

2 Aug 2026

Evaluating genetic diversity differences within a likelihood framework

PONE-D-25-04146R1

Dear Dr. Nickle,

We’re pleased to inform you that your manuscript has been judged scientifically suitable for publication and will be formally accepted for publication once it meets all outstanding technical requirements.

Within one week, you’ll receive an e-mail detailing the required amendments. When these have been addressed, you’ll receive a formal acceptance letter and your manuscript will be scheduled for publication.

An invoice will be generated when your article is formally accepted. Please note, if your institution has a publishing partnership with PLOS and your article meets the relevant criteria, all or part of your publication costs will be covered. Please make sure your user information is up-to-date by logging into Editorial Manager at Editorial Manager® and clicking the ‘Update My Information' link at the top of the page. For questions related to billing, please contact billing support.

If your institution or institutions have a press office, please notify them about your upcoming paper to help maximize its impact. If they’ll be preparing press materials, please inform our press team as soon as possible -- no later than 48 hours after receiving the formal acceptance. Your manuscript will remain under strict press embargo until 2 pm Eastern Time on the date of publication. For more information, please contact onepress@plos.org.

Kind regards,

Patrick R Stephens, Ph.D.

Academic Editor

PLOS One

Additional Editor Comments (optional):

Reviewers' comments:

Reviewer's Responses to Questions

**Comments to the Author**

Reviewer #1: All comments have been addressed

Reviewer #2: All comments have been addressed

Reviewer #3: All comments have been addressed

**********

2. Is the manuscript technically sound, and do the data support the conclusions?

Reviewer #1: Yes

Reviewer #2: Yes

Reviewer #3: Yes

**********

3. Has the statistical analysis been performed appropriately and rigorously?

Reviewer #1: Yes

Reviewer #2: Yes

Reviewer #3: Yes

**********

4. Have the authors made all data underlying the findings in their manuscript fully available?

The PLOS Data policy

Reviewer #1: Yes

Reviewer #2: Yes

Reviewer #3: Yes

**********

5. Is the manuscript presented in an intelligible fashion and written in standard English?

Reviewer #1: Yes

Reviewer #2: Yes

Reviewer #3: Yes

**********

Reviewer #1: (No Response)

Reviewer #2: I have no additional comments. The article "Evaluating genetic diversity differences within a likelihood framework" is well written.

Reviewer #3: I find the article is mainly on 'mathematics' rather than biology. However, the framework presented is for use in biology. I found the author did address all issues brought up by the two reviewers.

**********

what does this mean?). If published, this will include your full peer review and any attached files.

If you choose “no”, your identity will remain anonymous but your review may still be made public.

**Do you want your identity to be public for this peer review?**  For information about this choice, including consent withdrawal, please see our Privacy Policy

Reviewer #1: No

Reviewer #2: No

Reviewer #3: No

**********

# Editor report: Acceptance letter

**DOI:** 10.1371/journal.pone.0356079.r004

**Authors:** Patrick Stephens

PONE-D-25-04146R1

PLOS One

Dear Dr. Nickle,

I'm pleased to inform you that your manuscript has been deemed suitable for publication in PLOS One. Congratulations! Your manuscript is now being handed over to our production team.

At this stage, our production department will prepare your paper for publication. This includes ensuring the following:

* All references, tables, and figures are properly cited

* All relevant supporting information is included in the manuscript submission,

* There are no issues that prevent the paper from being properly typeset

You will receive further instructions from the production team, including instructions on how to review your proof when it is ready. Please keep in mind that we are working through a large volume of accepted articles, so please give us a few days to review your paper and let you know the next and final steps.

Lastly, if your institution or institutions have a press office, please let them know about your upcoming paper now to help maximize its impact. If they'll be preparing press materials, please inform our press team within the next 48 hours. Your manuscript will remain under strict press embargo until 2 pm Eastern Time on the date of publication. For more information, please contact onepress@plos.org.

You will receive an invoice from PLOS for your publication fee after your manuscript has reached the completed accept phase. If you receive an email requesting payment before acceptance or for any other service, this may be a phishing scheme. Learn how to identify phishing emails and protect your accounts at https://explore.plos.org/phishing.

If we can help with anything else, please email us at customercare@plos.org.

Thank you for submitting your work to PLOS ONE and supporting open access.

Kind regards,

PLOS ONE Editorial Office Staff

on behalf of

Dr. Patrick R Stephens

Academic Editor

PLOS One
